# Immune Checkpoint Inhibitors in Sinonasal Squamous Cell Carcinoma: A Retrospective Study and Literature Review

**DOI:** 10.3390/cancers17172872

**Published:** 2025-09-01

**Authors:** Kosuke Terazawa, Masashi Kuroki, Ken Saijo, Tatsuhiko Yamada, Ryota Iinuma, Ryo Kawaura, Hiroshi Okuda, Kenichi Mori, Hirofumi Shibata, Ryo Utakata, Miki Umeda, Takenori Ogawa

**Affiliations:** 1 Department of Otolaryngology-Head and Neck Surgery, Graduate School of Medicine, Gifu University, Gifu 501-1194, Japan; terazawa.kosuke.y4@f.gifu-u.ac.jp (K.T.); kurokim34@gmail.com (M.K.); yamada.tatsuhiko.a2@f.gifu-u.ac.jp (T.Y.); iinuma.ryota.h1@f.gifu-u.ac.jp (R.I.); kawaura.ryo.c9@f.gifu-u.ac.jp (R.K.); okuda.hiroshi.z1@f.gifu-u.ac.jp (H.O.); mori.kenichi.d6@f.gifu-u.ac.jp (K.M.); shibata.hirofumi.b3@f.gifu-u.ac.jp (H.S.); ray.allen3446@gmail.com (R.U.); miki.0215.shirokuro@gmail.com (M.U.); 2 Department of Clinical Oncology, Graduate School of Medicine, Tohoku University, Sendai 980-8575, Japan; ken.saijo.d6@tohoku.ac.jp

**Keywords:** head and neck cancer, sinonasal cancer, immune checkpoint inhibitor

## Abstract

**Objective**: Sinonasal squamous cell carcinoma (SNSCC) is a rare and aggressive malignancy, with limited treatment strategies in the recurrent or metastatic cases. Although immune checkpoint inhibitors (ICIs) have shown efficacy in head and neck cancers (HNCs), clinical data specific to SNSCC are scarce. This study aimed to evaluate the therapeutic efficacy and prognosis of ICIs in patients with SNSCC. **Methods**: We conducted a retrospective review of 18 patients with pathologically confirmed SNSCC treated with nivolumab or pembrolizumab at Gifu University Hospital between May 2017 and December 2024. Treatment response was assessed using RECIST v1.1 criteria. Overall response rate (ORR) and disease control rate (DCR) were evaluated as treatment effects, and overall survival (OS) and progression-free survival (PFS) were evaluated as prognoses. Subgroup analyses were performed according to treatment regimen. **Results**: The ORR and DCR for all patients were 43.8% and 56.3%, respectively. Pembrolizumab-treated patients showed higher response rates (ORR: 66.7%; DCR: 83.3%) compared to those treated with nivolumab (ORR: 30%; DCR: 40%). Median OS and PFS were 21.5 and 7.9 months, respectively. Long-term durable responses exceeding two years were observed in several cases. Although pembrolizumab tended to result in better outcomes, no statistically significant difference was found between groups. Immune-related adverse events were infrequent and manageable. **Conclusions**: This study suggests that a subset of patients with SNSCC may benefit from ICI therapy, particularly in combination with chemotherapy. Despite the rarity of SNSCC, accumulating clinical evidence—including prospective studies—is essential to establish standardized treatment strategies for this disease.

## Simple Summary

This retrospective study evaluated the therapeutic efficacy and prognosis of immune checkpoint inhibitors (ICIs) for recurrent or metastatic sinonasal squamous cell carcinoma (SNSCC). The overall response rate (ORR) was 43.8%, and the disease control rate (DCR) was 56.3%. The median overall survival (OS) was 21.5 months, and median progression-free survival (PFS) was 7.9 months. Notably, several patients experienced durable responses exceeding two years. These findings suggest that ICIs may offer therapeutic benefits for select SNSCC patients. Further prospective studies are essential to establish standardized treatment strategies for this rare malignancy.

## 1. Introduction

Sinonasal cancer is a malignant tumor that occurs in the nasal cavity and paranasal sinuses. It is a rare cancer that accounts for less than 5% of head and neck cancers (HNCs), and the most common primary site is the nasal cavity, followed by the maxillary sinus [1]. The pathological type is mostly squamous cell carcinoma (SCC), but compared to pharyngeal and laryngeal cancers, non-SCCs such as olfactory neuroblastoma and adenocarcinoma are also more common [2]. Sinonasal squamous cell carcinoma (SNSCC) is treated with multidisciplinary treatment including surgery, radiation therapy, and chemotherapy. SNSCC is often in an advanced stage at first diagnosis, and surgery is performed with extensive resection including surrounding tissue and reconstructive surgery to fill in the dead space [3]. In addition to radical chemoradiotherapy (CRT) after induction chemotherapy, radiotherapy and concomitant intra-arterial chemotherapy (RADPLAT) may also be used [4,5,6]. Despite these multidisciplinary treatments, SNSCC often has a poor prognosis, with a 2-year OS of 58.6% and a 5-year OS of 42.0% in cases of locally advanced cancer [4].

Traditionally, the treatment of SNSCC with recurrent or distant metastasis has consisted of a combination of platinum-based cytotoxic drugs and the molecular targeted drug cetuximab. However, evidence to date for chemotherapy of SNSCC with recurrence or distant metastasis is limited. Although the number of cases is small, a previous study of cases with recurrent sinonasal cancer, including SNSCC, showed that the median overall survival of cases treated with chemotherapy alone was 4.6 months [7]. Cases with clinical benefit after chemotherapy had a longer median OS than cases with disease progression (29.2 months vs. 4.4 months, *p* < 0.0001), although only a limited number of cases derived benefit.

In recent years, immune checkpoint inhibitors (ICIs) have become applicable for HNC, and ICIs have become a treatment option for SNSCC [8,9,10]. PD-1 (Programmed Death-1), an immune checkpoint receptor expressed on T cells, binds to its ligands PD-L1 and PD-L2 to inhibit T cell activation, thereby playing a role in preventing excessive inflammation and autoimmune responses, while also being exploited by cancer cells as an immune evasion mechanism [11]. Both nivolumab and pembrolizumab are human IgG4 monoclonal antibodies targeting PD-1; they competitively block ligand binding to PD-1 and thus reactivate T cell function [12,13]. CheckMate-141 and KEYNOTE-048, the clinical trials that provided evidence for ICIs in HNC, did not include SNSCC, so there is no evidence for ICIs in SNSCC [7,10]. Among the rare cancers of SNSCC, cases using ICIs are even rarer. However, the PD-L1 positivity rate in SNSCC is 34–46%, and it is believed that a certain number of cases will benefit from ICIs [14,15,16]. Moreover, high-density infiltration of CD8-positive tumor-infiltrating lymphocytes (TILs) within the tumor has been shown to be significantly associated with improved progression-free survival (PFS) and overall survival (OS), indicating that SNSCC is an immunoresponsive tumor and that inhibiting the PD-1/PD-L1 pathway may confer therapeutic benefit [14,16]. In this study, we retrospectively examined the therapeutic effect and prognosis of ICIs for SNSCC and reviewed previous reports.

## 2. Materials and Methods

### 2.1. Patients

This study included patients with SNSCC treated with ICIs (nivolumab or pembrolizumab) at Gifu University Hospital between May 2017 and December 2024. All cases were pathologically diagnosed as SCC. All relevant clinical data were obtained from patient medical records. Informed consent was not sought for this study because a waiver of consent was approved by the institutional review board due to an opt-out approach. This study was approved by the Institutional Ethical Committee of the Gifu University Graduate School of Medicine (2023-253) and was conducted in accordance with the Helsinki Declaration.

### 2.2. ICI Regimen

Nivolumab was selected for platinum-resistant cases where recurrence occurred within 6 months after treatment with platinum-based drugs. Nivolumab was administered at a dose of 240 mg every 2 weeks, and in stable patients at a dose of 480 mg every 4 weeks. Pembrolizumab was selected for patients with distant metastasis at first visit and for patients with platinum sensitivity; in short, recurrence occurred more than 6 months after the end of platinum-based therapy. Pembrolizumab was administered at a dose of 200 mg every 3 weeks, and at a dose of 400 mg every 4 weeks in stable patients. Pembrolizumab was administered in combination with chemotherapy based on PD-L1 expression level (CPS: Combined Positive Score). Biopsy and surgical tissue specimens were immunostained with an anti-PD-L1 mouse monoclonal antibody (Clone22C3), and the total number of positive cells, including not only tumor cells but also other lymphocytes, was divided by the total number of tumor cells. Cisplatin and 5-FU were used for combination drugs.

### 2.3. Data Collection and Endpoints

The following data were collected from medical records: age, sex, primary site, type of ICI, and immune-related Adverse Events (irAEs).

Therapeutic effect was evaluated using Response Evaluation Criteria in Solid Tumors (RECIST) version 1.1. Based on the best overall response (BOR) during the treatment period, the overall response rate (ORR) and disease control rate (DCR) were calculated. ORR was calculated as the percentage of cases that showed complete response (CR) and partial response (PR) among all cases, and DCR was calculated as the percentage of ORR plus stable disease cases. Prognosis was evaluated as overall survival (OS) and progression-free survival (PFS). OS was defined as the time from the date of first ICI administration to the date of death or last confirmed date. PFS was defined as the time from the date of first ICI administration to progressive disease (PD).

### 2.4. Statistical Analysis

Survival time analysis was performed using the Kaplan–Meier method, and differences between groups were analyzed using the log-rank test. Differences in therapeutic effect between the two groups were analyzed using the Chi-square test. All statistical analyses were performed using EZR (version 3.6.3). In all analyses, a *p*-value < 0.05 was considered significant.

## 3. Results

### 3.1. Patient Characteristics

A total of 18 cases of SNSCC were included (Table 1). The follow-up period ranged from 1 month to 77.7 months, with a median of 21.2 months. Ages ranged from 35 to 84, with a median age of 65, and 83.3% were male. The primary lesions were in the nasal cavity and maxillary sinus in 50% of cases for each. Nivolumab was used in 11 cases (61.1%), and pembrolizumab was used in 7 cases (38.9%). Of the seven cases in which pembrolizumab was used, five were administered in combination with chemotherapy. irAEs occurred in 5 cases (27.8%). There were no differences in patient characteristics between primary tumors.

### 3.2. Therapeutic Effect and Prognosis

**Figure 1:**
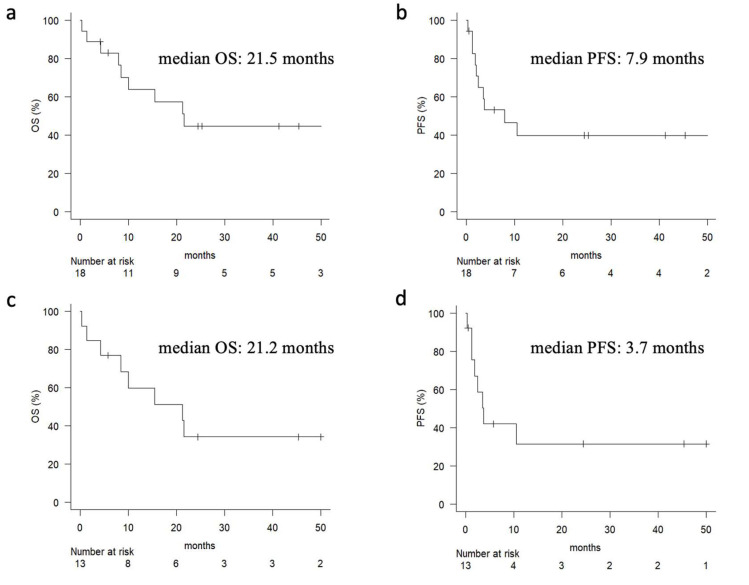
(**a**) Overall survival rate and (**b**) progression-free survival rate for all cases (*n* = 18) are shown. (**c**) Overall survival rate and (**d**) progression-free survival rate for ICI monotherapy cases (*n* = 13) are shown. PFS: progression-free survival, OS: overall survival.

**Figure 2:**
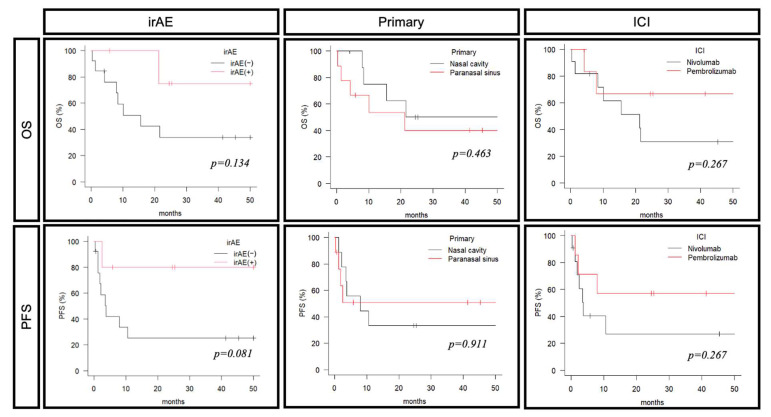
The difference in survival rate according to each factor (age, primary site, ICI) is shown. ICI: immune checkpoint inhibitor, OS: overall survival, PFS: progression-free survival.

Of all cases, three cases had CRs, four cases had PRs, two cases had SD, and seven cases had PD (Table 2). The ORR was 43.8% and the DCR was 56.3%, with many cases benefiting from the ICIs. When evaluated by drug, more cases achieved therapeutic benefit in the pembrolizumab group, which had an ORR of 66.7% and a DCR of 83.3%, compared to the nivolumab group, which had an ORR of 30% and a DCR of 40%. The median OS for all cases was 21.5 months, and the median PFS was 7.9 months (Figure 1a,b). In ICI monotherapy cases only, median OS was 21.2 months and median PFS was 3.7 months (Figure 1a,b). As with other HNCs, several cases showed long durable responses. irAEs were correlated with both the response rate and prognosis of ICI therapy. The ORR was 80.0% in patients who experienced irAEs, compared to 27.7% in those without irAEs (*p* = 0.0488). Remarkably, among the five cases who developed irAEs, two cases achieved CR and two cases achieved PR, suggesting a higher likelihood of favorable outcomes in this subgroup. Similar results were obtained for prognosis: cases with irAEs had better OS and PFS, but the number of cases was small and no significant difference was observed (OS: *p* = 0.134, PFS: *p* = 0.081) (Figure 2). Comparisons by age and primary site were made, but no significant differences were found. The prognosis tended to be better in the pembrolizumab group compared with the nivolumab group, but there was no significant difference.

### 3.3. Details of the Treatment Progress

**Figure 3:**
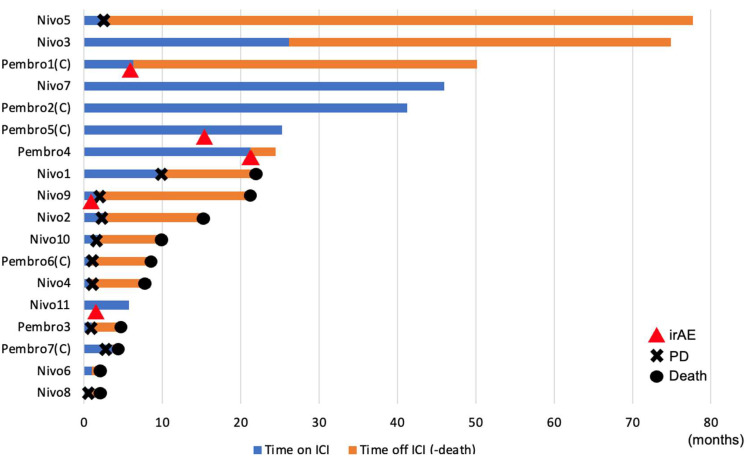
Swimmer plots of all cases are shown. Red triangles indicate irAEs, crosses indicate disease progression, and circles indicate death. Time on ICI indicates the period during which ICI was administered, and time off ICI indicates the period during which ICI was not administered. The (C) at the end of the case number indicates a chemotherapy combination case. irAE: immune-related adverse event, PD: progressive disease, ICI: immune checkpoint inhibitor.

The progress of all cases after ICI administration is shown in Figure 3.

irAEs occurred in 5 of 18 cases (27.7%). Two cases occurred in the nivolumab group and three in the pembrolizumab group: two cases of dermatitis, and one case each of adrenal insufficiency, pulmonary disorder, and colitis. The median duration of ICI treatment was 10.9 months (range: 1–46 months).

In the case of Nivo 5, the ICI resulted in PD, but the next treatment was effective, and the patient is living long-term without cancer. Of note, the cases of Nivo 3, Nivo 7, Pembro 2, and Pembro 5 achieved long-term responses lasting more than 2 years. In the case of Nivo 3, the ICI was discontinued at the patient’s request, and the patient is being observed without treatment, but there has been no recurrence for over three years. In addition, an irAE (colitis) occurred in the case of Pembro 1 six months after the start of ICI treatment, and the patient has not been treated since then, and there has been no recurrence for over three years.

## 4. Discussion

In this study, the therapeutic effect and prognosis of ICIs in SNSCC were investigated. It was shown that there are cases of SNSCC that benefit from ICIs, just like other types of HNC. Previous studies have demonstrated that SNSCC exhibits similar immunogenicity to other head and neck cancers, and our results may further support these findings [14]. In addition to our cases, we also reviewed previous reports. In addition to reports that have examined the therapeutic effect of ICIs in SNSCC alone, reports that have examined the therapeutic effect of ICIs in HNC, which clearly state the number of SNSCC cases and the therapeutic effect or prognosis of SNSCC alone, are summarized in Table 3 [17,18,19,20,21,22,23]. A total of 98 cases were included. The number of cases in each report was small, ranging from 4 to 18 cases, and our study had the largest number of cases for SNSCC alone. The ORR and DCR rates were 21.4% and 56.3%, respectively. In a retrospective study of SNSCC alone, similar to our study, Park JC et al. reported a small number of cases but an ORR of 27.2% and an mPFS of 4.2 months [18]. The ORR in clinical trials was 13.3–16.9%, which was comparable to other HNCs, although direct comparisons were not possible [8,9,10]. Regarding prognosis, the median PFS was 1.9–7.9 months, and the median OS was 7.7–27.7 months. It has also been shown that some SNSCC cases achieve durable responses, and these are phenomena specific to ICIs that are also seen in other cancers [24]. Durable responses have been observed not only in cases who continued ICI therapy but also in those who discontinued treatment after achieving a favorable response [25,26]. In our study, for instance, efficacy persisted for two years following cessation of the ICI in patient Nivo3, and patient Pembro1 achieved a durable response despite receiving only six months of the ICI. The precise biological mechanisms underlying this phenomenon remain incompletely understood, underscoring the need for further basic research and additional case analyses.

It is known that the occurrence of irAEs correlates with a good prognosis in many cancers [27,28]. Although there have been some reports examining the correlation between irAEs and prognosis in HNC, there have been no reports on SNSCC, and there is only one case report [29,30,31]. The case report was a 65-year-old man with SNSCC who had to discontinue ICI treatment several times due to the development of an irAE (pneumonia) after administration of pembrolizumab, but his disease held stable for more than 4 years [31]. The results of our study suggest that the occurrence of irAEs is associated with favorable response rates and prognoses in SNSCC, as in other HNCs. Furthermore, Pembro1 achieved a durable response despite the treatment being permanently discontinued due to irAEs.

One of the reasons why the pembrolizumab group had a better therapeutic effect and prognosis may be that our study included cases that were treated with chemotherapy. In 2025, the results of a clinical trial of pembrolizumab in combination with chemotherapy for R/M SNSCC were reported [32]. In this Phase II study, 20 patients with R/M SNSCC were treated with pembrolizumab in combination with nab-paclitaxel and cisplatin, and an excellent outcome was achieved, with an ORR of 60% and a DCR of 100%. These results are comparable to those seen in other R/M HNSCC cases and are expected to be applicable to clinical practice [33].

Recently, it has been reported that most nasal cavity tumors originate in the nasal vestibule, the most anterior part of the nasal cavity, with a completely different behavior from the other sinonasal malignancies [34,35]. Of the nine cases of nasal cavity cancer included in our study, none could be clearly determined to have originated in the nasal vestibule. Nasal vestibular carcinoma occurs in shallower areas compared to conventional nasal cavity cancers, so it is thought that it is often detected in an early stage. However, because this study focused on recurrent and metastatic cases that would be suitable for ICIs, there may not have been any cases of nasal vestibular carcinoma with a relatively good prognosis.

The limitations of this study are that it was a single-center retrospective study with a small number of patients. Second, a certain number of cases were included in the chemotherapy combination group, so the results may not reflect the therapeutic effect of ICIs alone. In fact, OS was almost the same, but PFS was better in the chemotherapy combination group. Third, the study period was long due to the small number of cases, and therefore the follow-up period varied widely. This variability may have affected survival analyses. SNSCC has many treatment options, including surgery, CCRT, RADPLAT, and heavy ion radiotherapy. However, there is a lack of standardized chemotherapy and prospective studies for R/M SNSCC, and in clinical practice, regimens including ICIs are selected as part of HNC. Because single-center studies have limitations in building evidence for rare tumor SNSCC, future multicenter prospective studies targeting ICI monotherapy cases are needed.

## 5. Conclusions

In this study, we investigated the therapeutic effect and prognosis of ICIs in SNSCC and reviewed previous reports. Although the number of cases is small, it was shown that there are a certain number of cases of SNSCC that benefit from ICIs. Furthermore, it was suggested that, like other cancers, the occurrence of irAEs in SNSCC correlates with good response rates and prognoses.

## Figures and Tables

**Table 1:** Patient characteristics.

Characteristics		Total (*n* = 18)	%	Nasal (*n* = 9)	Maxillary Sinus (*n* = 9)	
Age	Median (range)	65 (35–84)		64 (47–84)	68 (35–76)	
Sex	Male	15	83.3	7	8	*p = 0.5271*
	Female	3	16.7	2	1	
ICI	Nivolumab	11	61.1	5	6	*p = 0.6288*
	Pembrolizumab	7	38.9	4	3	
irAE	Yes	5	27.8	2	3	*p = 0.5987*
	No	13	72.2	7	6	

ICI: immune checkpoint inhibitor, irAE: immune-related adverse event.

**Table 2:** Therapeutic effect of ICIs.

	CR	PR	SD	PD	NE	ORR		DCR	
Nivolumab (*n* = 11)	1	2	1	6	1	30%	*p = 0.152*	40%	*p = 0.091*
Pembrolizumab (*n* = 7)	2	2	1	1	1	66.7%		83.3%	
Total (*n* = 18)	3	4	2	7	2	43.8%		56.3%	

CR: complete response, PR: partial response, SD: stable disease, PD: progressive disease, NE: not evaluable, ORR: overall response rate, DCR: disease control rate.

**Table 3:** Review of previous reports.

Reference No	Year	Author	Journal	*n*	Primary	Pathology	ICI	Response	Outcome
[17]	2020	Kim H	BMC Cancer	12	sinonasal	SCC	Pembro, Nivo	ORR: 18%, DCR: 63.6%	-
[18]	2020	Park JC	Oral Oncol	11	sinonasal	SCC	Pembro (8), Nivo (3)	ORR: 27.2%	mPFS: 4.2
[19]	2020	Sato Y	Cancer Manag Res	13	sinonasal	SCC (12), non (1)	Nivo	ORR: 23.1%, DCR: 30.8%	-
[20]	2021	Hanai N	Int J Clin Oncol	14	maxillary	SCC (11), non (3)	Nivo	ORR: 7.1%	mPFS: 1.9, mOS: 7.7
[21]	2022	Ueda Y	Oral Oncol	18	sinonasal	SCC (11), non (7)	Nivo	ORR: 6.7%, DCR: 46.7%	mPFS: 2.5, mOS: 27.7
[22]	2022	Nakano T	Anticancer Res	4	sinonasal	SCC	Pembro	ORR: 25%, DCR: 75%	-
[23]	2024	Zhang X	Am J Otolaryngol	8	sinonasal	SCC	Pembro, Nivo	-	1yOS: 62.5%, 1yPFS: 50%
Our cases				18	sinonasal	SCC	Pembro, Nivo	ORR: 43.8%, DCR: 56.3%	mPFS: 7.9, mOS: 21.5
Total				98				ORR: 21.4% (18/84), DCR: 50.9% (30/59)	

SCC: squamous cell carcinoma, ICI: immune checkpoint inhibitor, ORR: overall response rate, DCR: disease control rate, mPFS: median progression-free survival, mOS: median overall survival.

## Data Availability

The raw data supporting the conclusions of this article will be made available by the authors on request.
